# Cholera endemicity as a systems failure: a STAMP-CAST investigation in Kakuma and Dadaab refugee complexes in Kenya

**DOI:** 10.1186/s12889-026-28948-z

**Published:** 2026-08-07

**Authors:** James Oyoo, Annette Murunga, Kevin Otieno, Ben Ngoye

**Affiliations:** 1 Institute of Healthcare Management, Strathmore University Business School, Nairobi, Kenya; 2 Strathmore Institute of Mathematical Sciences, Nairobi, Kenya

**Keywords:** Cholera outbreak, WASH, STAMP-CAST, Refugees

## Abstract

**Background:**

Cholera outbreaks continue to pose a significant public health threat in Kenya and other low-and middle-income countries, particularly in humanitarian settings where overcrowding, inadequate water and sanitation services, population mobility, and climate-related events increase vulnerability. While conventional outbreak investigations identify epidemiological risk factors, they often provide limited insights into the systemic control and coordination failures that influence outbreak response. The study applied the Systems-Theoretic Accident Model and Processes - Causal Analysis based on Systems Theory (STAMP-CAST) to identify systems control structures, interactions, and feedback failures affecting cholera response in Kenyan refugee camps.

**Methodology:**

A cross-sectional mixed-methods design was employed, integrating both quantitative and qualitative data. The study was conducted at two refugee sites - Dadaab (Garissa County) and Kakuma (Turkana County) - in Kenya. Quantitative data were collected from 280 individuals who had recovered from cholera (172 in Dadaab and 108 in Kakuma), while qualitative data were obtained through 10 key informant interviews (KIIs) with stakeholders involved in cholera outbreak response. Quantitative data were analyzed using Stata, whereas qualitative findings were analyzed using the STAMP-CAST framework to examine system control structures, unsafe control actions, feedback mechanisms, and causal factors contributing to response failures.

**Results:**

STAMP-CAST identified multiple systemic control and feedback failures affecting cholera outbreak response across governance, coordination, surveillance, communication, and response management functions. Community health workers (CHWs) and county governments emerged as the principal sources of cholera-related information in both refugee settings. The systems analysis generated 22 recommendations targeting improvements in coordination, communication, surveillance, resource mobilization, and institutional learning to strengthen outbreak preparedness and response.

**Conclusions:**

The study demonstrates the value of STAMP-CAST as a systems thinking approach for analyzing cholera response in complex humanitarian settings. By identifying interacting control and feedback failures beyond those captured through conventional epidemiological analyses, the approach provides actionable evidence for strengthening governance, surveillance, coordination, and adaptive capacity, thereby improving resilience to future cholera outbreaks and other public health emergencies.

**Supplementary Information:**

The online version contains supplementary material available at https://doi.org/10.1186/s12889-026-28948-z.

## Introduction

Cholera remains one of the most significant waterborne infectious diseases worldwide, disproportionately affecting low- and middle-income countries (LMICs), particularly in Sub-Saharan Africa. An estimated 430 million people are at risk of cholera across Sub-Saharan Africa [1, 2]. Cholera, caused by Vibrio cholerae, is a waterborne bacterial disease that causes severe diarrhea and dehydration in humans and remains a significant threat to global health [3]. Annually, there are an estimated 1.3-4.0 million cases of cholera worldwide, resulting in between 21,000 and 143,000 deaths [4]. Humanitarian settings are especially vulnerable due to overcrowding, inadequate water, sanitation and hygiene (WASH) infrastructure, weak surveillance systems, and population displacements that facilitate rapid disease transmission and hinder timely outbreak detection and response [5].

The WHO-led Global Task Force on Cholera Control (GTFCC) Roadmap to 2030 seeks to reduce cholera mortality by 90% through integrated interventions including surveillance, case management, oral cholera vaccination, WASH, and community engagement [6, 7]. These concerted efforts renewed focus on the framework for synchronizing the efforts of countries, donors, implementing partners, and supporting the coordinated multisectoral implementation of cholera control measures [8]. The roadmap emphasizes three strategic priorities: (i) to focus on cholera hotspots in endemic countries with well-targeted interventions; (ii) to reinforce early detection and response to contain epidemics quickly; and (iii) to provide an effective mechanism for coordinated technical support, financing, and resources at the global and country level. These priorities are operationalized through five complementary pillars, including surveillance, case management, oral cholera vaccination (OCV), water, sanitation, and hygiene (WASH), and community engagement [9]. All the pillars cascade with each other and play an integral role in emergency responses and multisectoral responses in both the short-term and long-term [10]. Despite these coordinated efforts, recurrent outbreaks continue to occur in many endemic settings, suggesting that technical interventions alone are insufficient without effective coordination, governance, and system-wide response mechanisms.

Despite the concerted efforts by the GTFCC, the disease continues to claim the lives of people in Kenya, just like in many other Low- and middle-income countries (LMICs) [11, 12]. Recurrent outbreaks continue to occur in Kenya despite sustained improvements in WASH and public health interventions [13, 14]. Refugee settlements such as Kakuma and Dadaab are particularly vulnerable because outbreak response occurs within complex humanitarian systems involving multiple organizations, resource constraints, mass gatherings, and highly mobile populations [15, 16]. These interconnected drivers demonstrate that cholera is not solely the result of environmental exposure but emerges from complex interactions among health systems, governance structures, infrastructure, surveillance and feedback mechanisms, and community behavior, which influences outbreak preparedness and response [1, 17].

Even though cholera has been labeled as a predictable, preventable, and treatable disease, outbreaks continue to spread across the country, with a high number of mortalities estimated at 122 deaths as of March 2023 [6]. Effective cholera control depends not only on proven technical interventions but also on the effectiveness of system control structures, coordination mechanisms, communication pathways, and feedback processes [18, 19]. Failures in these interconnected functions can delay outbreak detection, impede coordinated action, and reduce the effectiveness of emergency response [20, 21]. While conventional epidemiological investigations are effective in identifying transmission pathways and population-level risk factors, they provide limited insight into how organizational interactions, communication channels, decision-making processes, and feedback mechanisms contribute to response failures during public health emergencies [15, 21].

Systems-thinking approaches have increasingly been applied to address complex public health challenges because they enable researchers to examine interactions among multiple actors and organizations rather than isolated events [7, 22]. Approaches such as causal loop diagrams, system dynamics, and realist evaluation have enhanced understanding of complex health systems by modelling dynamic relationships, feedback processes, and intervention effectiveness [23]. However, causal loop diagrams and system dynamics primarily focus on system behavior over time, while realist evaluation seeks to explain how and why interventions succeed or fail within specific contexts. These approaches provide limited guidance for systematically identifying failures in organizational control structures, decision-making processes, coordination mechanisms, and feedback pathways during emergency response [20]. In contrast, the STAMP-CAST extends traditional linear models of causation by viewing adverse outcomes as consequences of inadequate control, coordination, and enforcement of system constraints across multiple organizational levels [24]. Rather than attributing failures solely to individual actors or isolated events, STAMP-CAST examines how unsafe control actions, inadequate feedback mechanisms, and organizational processes interact to produce system-wide failures. These characteristics make STAMP-CAST particularly suitable for analyzing cholera outbreak response in humanitarian settings where multiple governmental and non-governmental organizations operate within complex socio-technical environments [25, 26].

Although systems-thinking methods are increasingly being applied in public health research to understand complex health system challenges, the application of STAMP-CAST to infectious disease outbreak response remains limited [27, 28]. To our knowledge, this study represents the first application of STAMP-CAST to analyze cholera outbreak response. While STAMP-CAST has been successfully applied in engineering, aviation, occupational safety, and patient safety, its application to cholera has not previously been reported [18]. Extending STAMP-CAST to infectious disease emergencies, this study demonstrates its value as a systems-thinking framework for identifying control failures, coordination challenges, and feedback deficiencies that influence cholera preparedness and response in humanitarian settings.

Accordingly, this study applies STAMP-CAST to examine cholera outbreak response in the Kakuma and Dadaab refugee settlements as a case study for demonstrating the application of a systems-theoretic approach to public health emergencies. Specifically, the study analyses system control structures, unsafe control actions, coordination failures, and feedback mechanisms to illustrate how STAMP-CAST can identify systemic vulnerabilities and inform more resilient outbreak preparedness and response systems.

## Methods

### Study design and settings

**Fig. 1:**
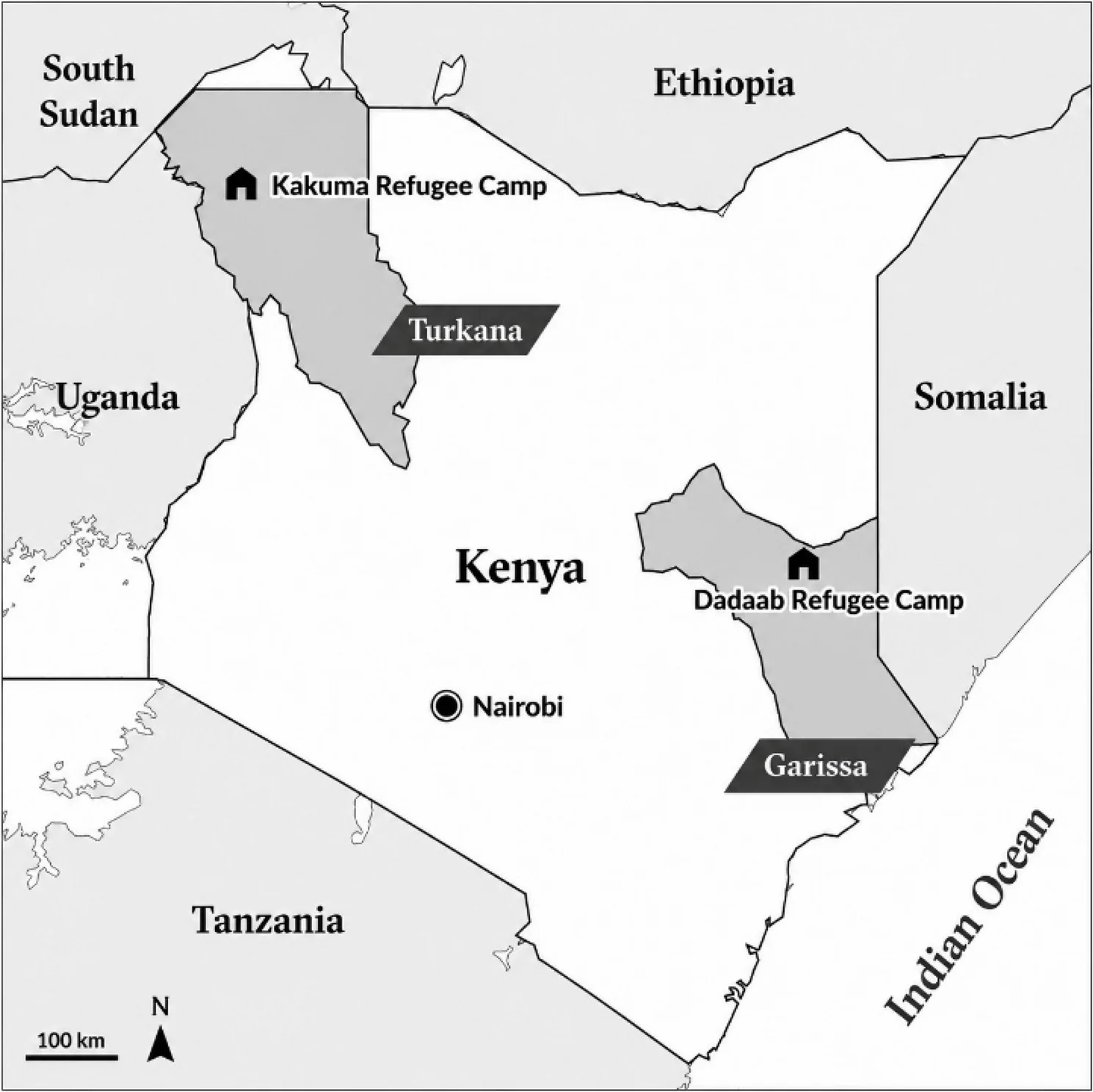
Map of Kenya showing Kakuma and Dadaab refugee settlements

A cross-sectional study comprising both qualitative and quantitative methods was conducted in two refugee zones in Kakuma and Dadaab refugee camps located in arid and semi-arid lands in the Northern part of Kenya [14, 29]. The camps were selected because they are humanitarian settings characterized by high population density, frequent population mobility, and recurrent cholera outbreaks, making them appropriate settings for examining system-level outbreak preparedness and response [30, 31]. Kakuma refugee settlement is in Turkana County; the settlement has a population of about 200,000 as of 2022, hosting refugees mainly from South Sudan, the DRC, Ethiopia, Rwanda, and Burundi. Dadaab refugee settlement is in Garissa County; the settlement has a population of 250,000 as of 2022, with refugees mainly from Somalia [29]. Some Cholera treatment centers (CTCs) within the settlements also serve the host communities. A total of 280 recovered cholera patients were interviewed, 172 from Garissa and 108 from Turkana, and a combined 10 key informant interviews were conducted in both counties as shown in Fig. 1 [14, 32].

### Study theoretical and analytical framework

**Fig. 2:**
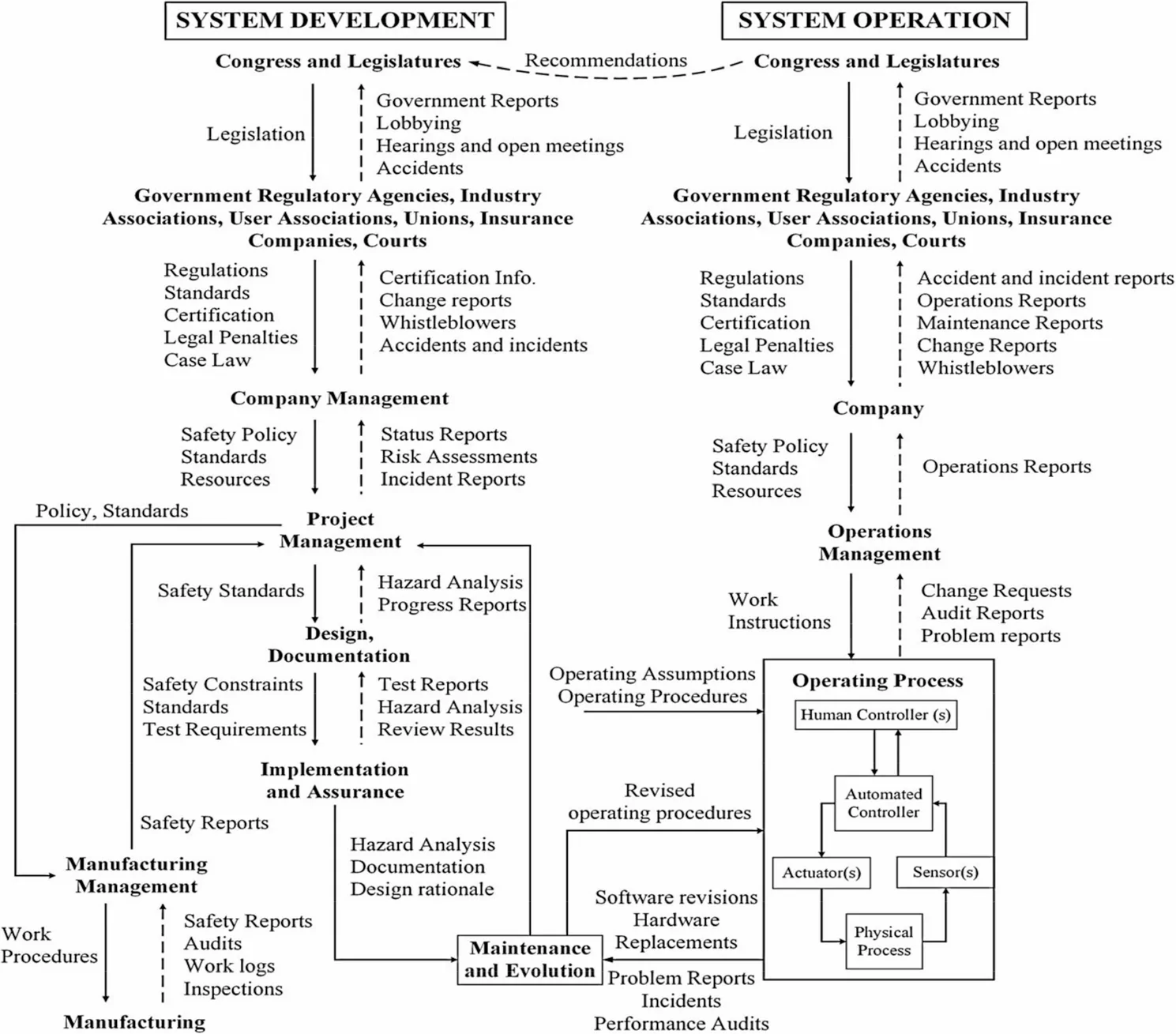
STAMP control structure model, adapted from [36]

Research on cholera has been predominantly epidemiological and primarily confined to the medical field, with a cause-and-effect approach to outbreaks. Considering the role played by disease surveillance systems, community engagement for raising awareness, health systems resilience, leadership, and coordination, among others, in cholera dynamics, an interdisciplinary approach should be adopted if the GTFCC strategic goal for 2030 is to be achieved [32, 33]. Therefore, we adopted a systems thinking approach, STAMP-CAST as a conceptual framework for this study, presented in Fig. 2, as it holds the promise to transcend the conceptual challenges noted above, in which studies on cholera are limited in their scope [34, 35].

Although no such work has yet been published in the literature, the method lends itself to the analysis of endemics and response, as the system representation it uses comprises two interconnected structures: development and operations. There are methods connected with STAMP that focus on accident (or event, in our case, cholera outbreak) analysis; however, STAMP does not produce accident models, but rather it produces system models [36]. We employed the STAMP-CAST framework in two primary ways: (1) to model the system as it current is, identify potential areas for failure, leverage points, and direct recommendations at specific system levels and actors; and (2) because it is not reliant on data from incidents, it can be utilized to highlight where things go right, allowing for a broader set of recommendations than analyses that can only proceed when (in this case) Cholera outbreak occurs, and where preparation and/or response was suboptimal.

STAMP-CAST: [37] is a systems-theory-based incident-analysis technique based on the premise that system failures (in our case, a cholera outbreak) result from insufficient control or enforcement of safety constraints and inadequate feedback mechanisms. It shifts the focus from human error alone to inadequate control structures, communication flaws, feedback failures, and unsafe interactions between system components. STAMP-CAST attempts to identify the dysfunctional interactions between each level of a system. The first step in constructing the STAMP-CAST model involves identifying the actors and organizations responsible for controlling the system. Control and feedback mechanisms between different system levels are then included to show what controls are enacted down the hierarchy and what information about the status of the system is sent back up the hierarchy [27, 38]

Actors Map: In this study, we employed the Rasmussen risk framework to develop the actor’s map. The Rasmussen Risk Framework helps identify actors by mapping decisions and behaviors across different levels of a socio-technical system. It considers government regulators, organizational managers, frontline workers, communities, and technology as interconnected layers that influence safety outcomes [39]. Each layer has its own pressures, constraints, and priorities, which shape how risk is managed. The framework shows how small shifts at one level can create unintended consequences elsewhere. This helps reveal where coordination, policy alignment, or capacity building are needed to reduce risk [40, 41].

We identified the actors in the STAMP-CAST control structure through a combination of document review, key informant interviews, and field observations. National and international cholera response documents, including the Kenya Ministry of Health cholera guidelines, the GTFCC roadmap, and humanitarian coordination frameworks, were reviewed to identify organizations with formal responsibilities for cholera preparedness, surveillance, case management, coordination, and response [39, 42]. These findings were complemented by information obtained from key informants, who described organizational roles, reporting relationships, communication pathways, decision-making processes, and interactions among stakeholders during cholera outbreak response [43].

**Fig. 3:**
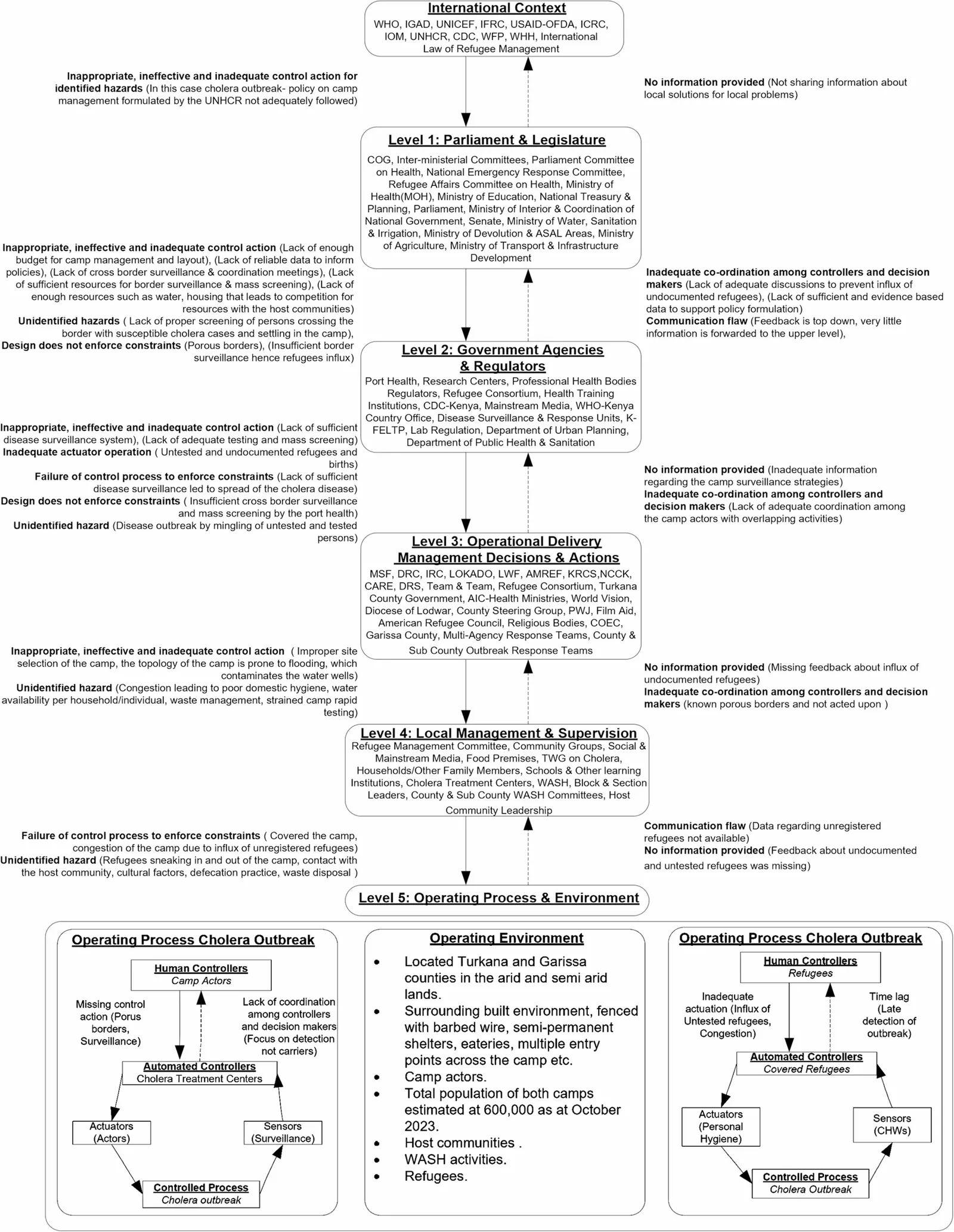
Kakuma and Dadaab refugee camps cholera endemic STAMP-CAST model

Consistent with the STAMP-CAST systems approach, the analysis included actors operating across the entire outbreak response system rather than only those directly involved in patient care [38, 44]. Actors were therefore mapped across hierarchical system levels, including international organizations, national and county governments, humanitarian agencies, health facilities, and community-level actors [45, 46]. This approach enabled the identification of control actions, feedback mechanisms, and organizational interactions that influenced cholera preparedness and response, providing the foundation for constructing the system control structure and identifying system-level control and feedback deficiencies [15]. The complete actor maps developed during system characterization are provided in Additional file 1 (Supplementary Figures S1–S2). These maps informed the construction of the STAMP-CAST control structure presented in Fig. 3.

After graphically plotting the responsible organizations at various levels of the system, the relevant control and feedback constraints were annotated on the control structure with solid downward arrows representing control mechanisms and dashed upward arrows representing feedback mechanisms. Each of the systematic elements identified within the control structure was then classified according to the classification of the flawed control [37, 38]. This taxonomy functions by evaluating the potential contribution of each control and feedback loop, listing the potential failures which are presented in Table 1. These are general factors applicable at each level of the control structure, but the interpretations of the factors will differ based on their application at different levels [47].

**Table 1:** Taxonomy of control flaws resulting in possible hazards

1. Inadequate or inappropriate control actions issued by the controller	i) Failure to identify possible hazards. ii) Inadequate coordination among controllers and decision-makers.
2. Inadequately executed control actions	i) Communication flaws ii) Time lags iii) Inadequate actuator operation
3. Inadequate or missing feedback	I) Time lag II) Communication flow III) Non-existent feedback process IV) Inadequate coordination among controllers and decision-makers

Using the taxonomy of control flaws resulting in hazards as the guiding framework for examining cholera response performance in Kakuma and Dadaab refugee settings. We examined (i) the control actions issued at each level (e.g., outbreak declaration, WASH activation, risk communication), and (ii) the feedback loops informing those actions (e.g., surveillance data, case trends, community alerts) [47]. This analytic approach enabled systematic identification of where control structure failures occurred and how they contributed to response inefficiencies [45]. Table 2 below shows how control structures, control actions, feedback loops, and system-level failures were used to analyze response inefficiencies.

**Table 2:** Conceptual Mapping of STAMP-CAST concepts to cholera response in Kakuma and Dadaab refugee settings

STMP-CAST Framework	Meaning in Accident Theory	Translation to Cholera Control
System Loss Event	Accident or unintended harmful outcome	Cholera outbreak or sustained transmission
Control Structure	Hierarchy of actors who issue constraints to maintain safety	Governance and coordination structures across national, county, facility, NGO, and community levels
Control Actions	Directives meant to maintain a safe system state	Outbreak declaration, WASH deployment, case management protocols, surveillance alerts
Feedback Loops	Information flows that allow adjustment of control actions	Sharing real-time surveillance data, field reports, IPC audits, and community alerts
Control Structure Failure	Breakdowns in constraints, communication, or authority	Delayed response activation, surge capacity, fragmented coordination, and inconsistent SOP adherence

### STAMP-CAST analytical process

Following construction of the system control structure, actor maps, interview transcripts, documentary evidence, and quantitative survey findings were systematically coded using the predefined STAMP-CAST taxonomy of control actions, feedback mechanisms, and control flaws [48]. Coding was conducted independently by two researchers, with differences in coding and interpretation resolved through consensus discussions [49]. Where agreement could not be reached, the Principal Investigator reviewed the evidence and made the final determination to ensure consistency in the application of the STAMP-CAST framework.

Identified control flaws were subsequently grouped according to the relevant level of the outbreak response system (national, county, humanitarian agencies, health facility, and community) and synthesized into broader system-level control and feedback deficiencies [39]. The analysis identified 17 inadequate control actions, 6 communication failures, 5 coordination failures, and 7 feedback deficiencies across the outbreak response system, giving a total of 37 control flaws. These synthesized deficiencies formed the basis for developing the 22 system-level recommendations presented in the discussion. Recommendations were not formally prioritized; rather, each recommendation was explicitly linked to one or more identified control or feedback deficiencies within the STAMP-CAST model.

Quantitative survey findings were integrated throughout the STAMP-CAST analysis to strengthen interpretation of the identified system failures [27]. Survey findings on sources of cholera information, access to WASH services, care-seeking behavior, and resource availability were triangulated with qualitative evidence to confirm or refine the identification of control failures, coordination breakdowns, and feedback deficiencies [48]. Areas of convergence strengthened confidence in the identified system weaknesses, while divergent findings prompted further examination of the qualitative data before finalizing the STAMP-CAST model.

The preliminary STAMP-CAST model was iteratively reviewed by the research team to ensure consistency between the empirical findings and the proposed control structure. The final model was subsequently reviewed and approved by the Principal Investigator and is presented in Fig. 3.

### Sampling and sample size

The study implemented an exhaustive sampling approach of the cases that occurred in Kakuma and Dadaab refugee settlements during the last outbreak between September and October 2018 and in 2021 [30]. The target population was adults above 18 years of age in Kakuma and Dadaab refugee settlements. An informed consent form was administered to the study participants; the participants were assured of no repercussions if they chose not to participate in the study [50, 51]. Consequently, for quantitative data, the responses were based on the nature and severity of illness for those affected, and from those who provided the services to those affected. Participants for the qualitative aspects of the study were purposively selected to represent the breadth of system actors involved in cholera preparedness and response, in line with the requirements of the STAMP-CAST framework [52]. These included patients, caregivers, frontline responders, institution managers, and policymakers. Additionally, 10 KII in both Dadaab and Kakuma refugee settlements were interviewed by the research team, whose perspectives informed the systems analysis of control structure failure and feedback breakdowns [53].

### Data collection tools and procedures

The study used both primary and secondary data sources [14, 53]. The secondary information was mainly obtained from written records and archived materials [54]. The primary data set was collected by employing multi-level data collection techniques. It is believed that combining two methods enriches the research by diversifying sources of information and triangulation of information sources, where findings in one method will be checked or supported against others [14, 54] to maximize the benefits of each method by compensating for the weakness of each method, and to establish confidence and obtain trustworthiness of the research findings. The tools used for primary data collection in this study were the household survey and Key informant interviews (KII) conducted by the research team [55].

### Household survey

A household survey was used to capture quantitative data on respondents’ demographics and clinical characteristics. We employed a standard survey instrument to collect information from a total of 280 cholera recovered patients who were interviewed, 172 from Dadaab and 108 from Kakuma. Ten community health workers were recruited and trained on the objectives of the study, the data collection tools, and the data collection software (Kobo Collect) [54]. The survey tool was prepared in English.

Trained supervisors checked the work of data collectors daily at the back end of the Kobo Collect tool for completeness and data quality [33]. The data were sent to the centrally shared drive, where completeness and quality were checked immediately. The field supervisor was responsible for ensuring that the data collectors followed the protocol to correct any errors that may have arisen during the administration of the questionnaire.

### Data analysis

#### Quantitative analysis

Survey data were analyzed using Stata version 17. Descriptive statistics (means, standard deviations, and proportions) were used to summarize participant characteristics and WASH-related practices [51]. Chi-square tests of independent was used to assess significant differences across the two settings (Kakuma and Dadaab) with significance set at p < 0.05. The quantitative analysis was not intended to establish causal relationships independently but to provide empirical evidence that informed and validated the subsequent STAMP-CAST systems analysis [51].

#### Qualitative analysis

Interview and observational data were coded and analyzed thematically using a hybrid deductive-inductive approach [21, 52]. An initial codebook was derived from the STAMP-CAST framework (control structures, control actions, feedback loops, and control structure failures), followed by inductive refinement to capture context-specific response dynamics. Coding was conducted independently by two researchers, with discrepancies resolved through consensus.

### Data triangulation

Quantitative findings from the survey were used to characterize baseline WASH access, health-seeking behaviors, and outbreak awareness patterns [33, 34]. These results informed the interpretation of system control failures identified through the STAMP-CAST analysis. Specifically, frequency distributions and cross-tabulations from the survey were compared against qualitative accounts of response coordination and operational constraints, enabling the identification of converging or divergent patterns across control-action levels. The STAMP-CAST model was therefore not applied in isolation but interpreted in relation to empirical observations on resource gaps, communication patterns, and implementation bottlenecks across the study settings.

Survey findings directly informed the interpretation of several STAMP-CAST components. For example, quantitative evidence identifying community health workers as the primary source of cholera information reinforced their role as key controllers within the system control structure. Similarly, survey findings related to delays in care-seeking, access to WASH services, and communication channels were triangulated with qualitative evidence to identify control failures, feedback deficiencies, and coordination gaps. Where quantitative and qualitative findings converged, confidence in the identified system failures was strengthened. Any divergent findings were re-examined during the STAMP-CAST analysis to refine the interpretation of system control and feedback mechanisms.

Recommendations were developed by linking each identified control flaw to the corresponding control structure responsible for mitigating that failure. Recommendations were not formally weighted or prioritized; rather, they were organized according to the system level at which intervention would most effectively strengthen outbreak preparedness and response [35, 56].

## Results

The sections below present site-specific findings for Kakuma and Dadaab, followed by a synthesis of shared patterns and contextual differences.

### Socio-demographic characteristics

In Kakuma (Turkana County), 108 recovered cholera patients participated in the study. Participants had a mean age of 27.0 years (SD 13.0; range 2–56 years), 60% were male, and 50% had attained basic education. In Dadaab (Garissa County), 172 recovered cholera patients participated. Participants had a mean age of 38.0 years (SD 15.0; range 5–73 years), 66% were female, and 48% had attained basic education. The difference in mean age between participants in Kakuma and Dadaab was statistically significant (Mean Difference = 10.042, *t* = 5.513, *p* < 0.05). These reflect the underlying demographic and social characteristics of the two refugee settlements.

### Cholera knowledge and community perceptions

Awareness of cholera was high in both sites (Kakuma 98%; Dadaab 97%), with community health workers identified as the main source of cholera-related information. In Kakuma, respondents most frequently associated cholera with drinking contaminated water (26%) and eating contaminated food (19%), while in Dadaab, poor hygiene (24%) and contaminated water (24%) were the most commonly reported causes. Open defecation was cited in both settings (18%) as shown in Table 3. The findings also reveal that there were no significant differences between the two sites (*χ² = 8.012*, *p* = 0.2372 > 0.05).

**Table 3:** Respondents’ awareness of the causes of cholera

What causes cholera?	Kakuma (%)	Dadaab (%)
Drinking contaminated water	26	24
Eating contaminated food	19	16
Unwashed fruit/vegetables	3	4
Flies and other Insects	15	15
Poor hygiene	18	24
Open defecation	18	18
χ² = 8.012, *p* = 0.2372		

Across both sites, seeking urgent medical attention was the most frequently reported response when experiencing cholera symptoms, cited by 26% of respondents in Kakuma and 38% in Dadaab. Boiling or treating drinking water was the next most common action (18% in Kakuma; 19% in Dadaab). Use of a latrine/avoiding open defecation (12% Kakuma; 13% Dadaab) and general food and utensil hygiene practices, such as covering food, cleaning utensils, and cooking food thoroughly, were also reported, though at relatively lower frequencies and with slight variation between sites. A small proportion reported using herbal remedies (2% Kakuma; 3% Dadaab). Only 1% in both settings reported that there was nothing one could do in the event of cholera, indicating generally high perceived agency in response actions. These are illustrated in Table 4 below.

**Table 4:** Respondents perception of what should be done if they have cholera

What should one do if they have cholera?	Kakuma (%)	Dadaab (%)
Seek urgent medical attention	26	33
Boil or treat drinking water	18	19
Use a latrine/Avoid open defecation	12	13
Clean cooking utensils	11	10
Cook food thoroughly	11	7
Cover food	9	6
Reheat stored food	8	4
Wash vegetables and fruit	3	5
Use herbal remedies	2	3
There is nothing one can do	1	1
χ² = 5.949, *p* = 0.745		

### Health-sector response to cholera outbreaks

Most respondents reported receiving cholera sensitization during the outbreak from county authorities (Kakuma 98%; Dadaab 98%). In Kakuma, 87% noted that the response was activated within one day to one week, compared to 54% in Dadaab. The nature of the response varied across the two settings: public health education and hygiene promotion was the most frequently reported action (Kakuma 58%; Dadaab 52%), followed by strengthening of health facilities to accommodate increased caseloads (Kakuma 8%; Dadaab 23%). Provision of free or subsidized healthcare was also noted (Kakuma 20%; Dadaab 15%), while procurement of medical supplies was less frequently reported (Kakuma 3%; Dadaab 6%). Temporary closure of public eating places was uncommon in both settings (Kakuma 8%; Dadaab 4%) as illustrated in Table 5. Kakuma and Dadaab respondents differ significantly in their perceptions of the nature of the response to cholera (*χ² = 13.239*, *p* = 0.0212 < 0.05).

**Table 5:** Nature of the health sector response in case of a cholera outbreak

Nature of health sector response	Kakuma (%)	Dadaab (%)
Public health education and hygiene promotion	58	52
Provision of free or subsidized healthcare	20	15
Strengthening of health facilities to receive more people	8	23
Closure of public eating places	8	4
Procurement of medical supplies and equipment	3	6
Don’t know	3	1
χ² = 13.239, *p* = 0.0212		

### Care-seeking behavior during cholera outbreaks

In Kakuma, respondents mainly sought treatment at government hospitals (44%) and dispensaries/health centers (36%), while community health workers were reported as a source of care by 12% of respondents. In Dadaab, respondents similarly reported seeking care mainly from hospitals/government facilities (38%), dispensaries/health centers (35%), and community health workers (21%), as shown in Table 6.

**Table 6:** Healthcare-seeking behavioral practices during cholera outbreaks

Treatment Source	Kakuma (%)	Dadaab (%)
Hospital / Government facility	44	38
Dispensary / Health center	36	35
Community health worker	12	21
Private clinic	3	1
Chemist	2	2
Traditional healer	1	3
Family, Neighbor, Kiosk/Shop	1	0
χ² = 8.012, *p* = 0.2372		

The quantitative findings provided evidence on community knowledge, care-seeking behavior, and perceptions of outbreak response, while the qualitative findings explained the organizational and system factors underlying these observations. Integrating these datasets within the subsequent STAMP-CAST framework enabled the identification of system control actions, communication pathways, feedback deficiencies, and coordination failures that contributed to the overall outbreak response.

### Interpretation of quantitative findings

Although both refugee settlements demonstrated high levels of cholera awareness and use of formal healthcare services, response performance differed between sites. In Kakuma, 87% of respondents reported that outbreak response activities were initiated within one day to one week, compared with 54% in Dadaab. Similarly, respondents in Kakuma more frequently reported receiving free or subsidized healthcare, whereas respondents in Dadaab more often highlighted efforts to expand health facility capacity. The STAMP-CAST analysis suggests that these differences reflect variations in system-level coordination, surveillance, communication, and resource mobilization across the two settings.

### STAMP-CAST analysis of system control and feedback failures

#### Construction of the STAMP-CAST control structure

The quantitative differences observed between Kakuma and Dadaab were further explained through the STAMP-CAST analysis, which identified control and feedback deficiencies across multiple organizational levels involved in cholera outbreak response [18, 48]. These deficiencies included weaknesses in governance, surveillance, cross-border coordination, communication, resource allocation, and camp management [57, 58]. Figure 3 illustrates the system control structure, while a detailed mapping of individual STAMP-CAST findings to the 22 system-level recommendations is provided in Table 7 [59].

#### Identified unsafe control actions and control failures

The analysis identified several control failures affecting outbreak preparedness and response. At the international level, gaps were identified in the implementation and enforcement of camp management policies [25]. At governance levels, insufficient resource allocation, weak surveillance systems, limited cross-border coordination, and inadequate data feedback mechanisms affected response capacity.

At operational levels, infrastructure limitations, overcrowding, inadequate WASH capacity, and limited emergency preparedness constrained effective outbreak control [60, 61]. At community and local management levels, population movement, communication challenges, and limited stakeholder engagement affected implementation of preventive measures [61, 62].

#### Feedback deficiencies and coordination failures

Analysis of feedback pathways identified gaps between surveillance systems, decision-makers, humanitarian actors, and communities [25, 61]. Delays in information flow, inconsistent reporting mechanisms, and limited coordination between actors reduced the ability of the response system to rapidly adapt to emerging outbreak conditions [63].

#### System vulnerabilities identified through STAMP-CAST

The STAMP-CAST model demonstrated that cholera transmission was influenced by interacting vulnerabilities across multiple system levels rather than isolated failures of individual actors [62, 64]. These vulnerabilities included governance constraints, WASH, weak surveillance feedback loops, infrastructure limitations, and challenges in coordinating multi-sectoral response activities [18, 63].

**Table 7:** Detailed STAMP-CAST findings and associated recommendations (R)

System Level	Identified Control/Feedback Failure	System Interpretation	Recommendation
International Context	UNHCR camp management policies were not consistently implemented.	Weak enforcement of international governance reduced accountability and consistent implementation of camp management standards across humanitarian actors.	R1: Implement UNHCR policies and guidelines as stipulated.
Parliament & Legislature	Inadequate budget allocation for camp management and layout.	Insufficient financial control limited preparedness and response capacity.	R2: Establish contingency funds to respond to emergencies within the camps.
	Limited reliable data to inform policy decisions.	Weak strategic feedback mechanisms constrained evidence-informed policymaking.	R3: Strengthen national data systems to support evidence-informed policy development.
	Insufficient resources for border surveillance and mass screening.	Resource constraints reduced the effectiveness of surveillance and early case detection.	R4: Increase investment in border surveillance and mass screening.
	Porous borders allowed entry of untested refugees.	Weak border control increased the likelihood of cross-border disease transmission.	R5: Strengthen border management through one-stop border posts and coordinated screening.
Government Agencies & Regulators	Disease surveillance systems were insufficient.	Weak surveillance delayed outbreak detection and situational awareness.	R6: Strengthen disease surveillance systems.
	Inadequate testing and mass screening capacity.	Limited diagnostic capacity reduced timely case identification.	R7: Improve testing and mass screening capacity.
	Untested and undocumented refugees and births.	Weak registration systems created surveillance blind spots.	R8: Strengthen refugee registration and routine health screening.
	Delayed notification following disease detection.	Inadequate feedback between surveillance and decision-makers delayed response.	R9: Strengthen immediate case identification and notification systems.
	Weak cross-border surveillance and coordination.	Poor interagency coordination limited information sharing and joint response.	R10: Conduct regular cross-border surveillance and coordination meetings.
	Mixing of tested and untested populations.	Failure to implement infection prevention controls increased transmission risk.	R11: Strengthen adherence to MOH cholera response guidelines and improve outbreak control.
	Limited coordination among response actors and inconsistent reporting.	Fragmented governance reduced coordinated decision-making and operational effectiveness.	R12: Integrate emergency response under one coordination mechanism, establish regular Technical Working Group meetings, and standardize data collection and reporting.
Operational Delivery, Management Decisions & Actions	Camp sites were located in flood-prone areas that contaminated water sources.	Poor infrastructure planning increased environmental exposure to cholera.	R13: Strengthen existing infrastructure and establish safer camp sites where appropriate.
	Inadequate water supply and housing created resource competition.	Resource shortages reduced system resilience and increased vulnerability.	R14: Increase water supply and improve camp infrastructure.
	Congestion, inadequate sanitation, limited testing capacity, and insufficient toilets.	Weak operational control and inadequate WASH capacity increased transmission risk.	R15: Coordinate WASH actors under a unified operational structure.
	Emergency response teams were not consistently prepared.	Operational readiness was insufficient for rapid outbreak response.	R16: Ensure response teams are identified and readily deployable.
	Limited availability of emergency supplies and diagnostic kits.	Weak logistical preparedness constrained timely interventions.	R17: Maintain emergency supplies and cholera testing kits.
	Inadequate nutrition support during outbreaks.	Nutritional vulnerabilities reduced resilience among affected populations.	R18: Improve nutrition and food supplies during outbreaks.
	Limited capacity of Community Health Workers.	Community-level control actions were constrained by insufficient training.	R19: Train Community Health Workers in health promotion and cholera case management.
Local Management & Supervision	Congestion due to influx of unregistered refugees.	Weak local supervision limited surge management and preparedness.	R20: Strengthen surge capacity and ensure early preparation of essential supplies.
	Population movement, cultural practices (communal gatherings, funeral practices), poor waste disposal, and limited community engagement.	Weak community-level control and feedback reduced compliance with preventive measures.	R21: Intensify communication, social mobilization, community engagement, and routine water quality monitoring.
	Budgetary constraints and weak supervision of operational activities.	Limited local oversight reduced effective implementation and accountability.	R22: Strengthen supervision of health commodities and stakeholder engagement, including religious leaders, in camp management.

## Discussions

Response to cholera outbreaks among populations affected by complex humanitarian crises can be a daunting task comprising multiple interacting moving components that are both applied in urban and rural contexts. This study employed a mixed method approach comprising qualitative and quantitative methods. We then applied a systems-based approach by utilizing the STAMP-CAST method which generated a total of 22 recommendations to complement the quantitative analysis. The package of interventions by the various actors varied but common challenges were identified. The findings from this study are discussed in relation to the following key areas in the Kenyan context: (a) Health System Structures and Interactions, (b) Identification of Weaknesses within the Health System, (c) Recommendations for System Optimization within the Health System (d) Resilience and Adaptability of the Health System.

### System structures and interactions within the health systems

Overall, the coordination of cholera interventions was very good at both the National, County, and sub-county levels in the current study. Nonetheless, camp surveillance and community engagement interventions were weak in terms of frequency and resource allocation. It is common for national public health authorities to have well-developed policies and legislative frameworks for governing outbreak response that are not matched with the actual implementation at sub-county and community levels [65]. In retrospect, other coordination indicators such as the availability of a dedicated cholera technical working group (TWG) teams, roles and responsibilities of TWGs, mobilization and allocation of resources for preparedness, simulation exercises, and pre-positioning of essential supplies were found to be inadequate in this study. Therefore, strengthening coordination mechanisms for response teams, heightening surveillance and enhancing surge capacity for rapid testing of returning and new refugees who are susceptible to cholera, and having cross-border coordination meetings are expected to respond to and contain the spread of cholera disease.

### Identification of weaknesses within the health system

According to the [66], ensuring access to safe water and sanitation, promoting good hygiene practices, and administering Oral Cholera Vaccines (OCVs) are some of the best practices for preventing cholera. Experiences from COVID-19 have highlighted the importance of increased capacity for prediction, preparedness, and early response in controlling and reducing mortality [67]. Drawing from these experiences in the context of cholera, well-defined communication protocols, coordination meetings even if there is no cholera outbreak, observing mitigation orders, provision of supplies and contact tracing plays a big role in alleviating the spread of cholera during the outbreaks [1].

### System optimization within the health system

Listening to and responding to public inputs and feedback is essential for strengthening health systems and ensuring fairness in healthcare access. Responsiveness fosters a more inclusive and effective system where policies and practices align with community needs. This concept is closely linked to citizen, public, or community participation in health systems, which enhances accountability, trust, and service delivery [68, 69]. Our findings suggest that more attention should be given to training CHWs more on community engagement and cholera case management strategies, harnessing the strengths of different actors which will in turn help with avoiding role replication, strengthening the existing infrastructure as per the MOH guidelines and GTFCC, improving communication flow in a top-down-bottom-up approach among the stakeholders. However, in our context, a key question remains on how to approach the host communities and the refugees respectfully and treat them as essential stakeholders in the design and implementation of interventions targeting cholera control.

#### Resilience and adaptability of the health system

According to the WHO, cholera epidemics occur mostly in Sub-Saharan Africa. In the Kenyan context, population mobility associated with complex humanitarian emergencies can increase the risk of cholera transmission when people settle in overcrowded environments with limited access to safe water, sanitation, disease surveillance, and healthcare services [70]. These findings underscore the importance of resilient health systems, coordinated cross-border surveillance, and strengthened humanitarian preparedness rather than the movement of populations itself [57]. These events, coupled with the threats of climate change and flash floods, require resilient and adaptable health systems that can deal with these unlikely events. Nevertheless, the camp actors need to align and complement their efforts under one roof for ease of response in case of cholera outbreaks, and to develop strategies that will prevent cholera spread within the camp setting [70].

To our knowledge, our study is the first comprehensive assessment of cholera outbreak and response using the STAMP-CAST methodology to implement multi-stakeholder cholera interventions in a unique, fragile, and cholera-endemic setting. This is an important strategy for strengthening Kenya’s capacity and other cholera-endemic countries to actualize the GTFCC’s roadmap goals by 2030 given the unique setting in which the study was carried out. Further, the study provided evidence in the context-specific gaps in the multi-stakeholder cholera interventions that should be prioritized for strengthening. Our study has also made methodological contributions of public health importance, particularly in the disaster risk management field.

### Recommendations

The STAMP-CAST analysis identified system-level control and feedback deficiencies spanning governance, surveillance, operational delivery, and community engagement. Based on these findings, the following recommendations are proposed to strengthen cholera preparedness and response in refugee and other humanitarian settings.

### Governance and coordination

The findings highlight the need for stronger governance mechanisms to improve coordination among government agencies, humanitarian organizations, and community stakeholders. Strengthening institutional accountability, ensuring sustained financing for outbreak preparedness, and establishing regular multisectoral coordination mechanisms before outbreaks occur would enhance the effectiveness of emergency response [64, 71]. Improved implementation of existing national and international guidelines, including those of the Ministry of Health and UNHCR, would further support coordinated outbreak management.

### Surveillance and information systems

Early outbreak detection depends on timely surveillance, efficient reporting, and effective feedback between frontline health workers and decision-makers. Strengthening integrated disease surveillance systems, standardizing reporting tools, improving laboratory capacity, and enhancing cross-border surveillance and information sharing would facilitate earlier identification of cholera cases and more rapid implementation of control measures [1, 59].

### Community engagement

Community health workers, local leaders, and humanitarian partners were identified as important actors in cholera prevention and response. Strengthening community engagement through continuous health promotion, culturally appropriate risk communication, and structured community feedback mechanisms would improve early reporting of suspected cases, increase adherence to preventive measures, and strengthen trust between communities and response agencies [70, 72].

### WASH and infrastructure

The STAMP-CAST analysis identified infrastructure limitations, overcrowding, inadequate water and sanitation services, and shortages of emergency resources as important system vulnerabilities. Investments in resilient WASH infrastructure, emergency preparedness planning, pre-positioning of essential supplies, and strengthening the capacity of frontline health workers would improve the ability of the health system to respond effectively to future outbreaks [64].

### Cross-border coordination

Given the frequent movement of populations across international borders, cholera preparedness requires sustained collaboration between neighboring countries and humanitarian agencies. Strengthening cross-border surveillance, harmonizing reporting systems, and conducting joint preparedness and response activities would improve coordination and reduce the risk of cross-border transmission during outbreaks [57, 61].

These recommendations emphasize strengthening the control structure of cholera outbreak response by improving governance, surveillance, communication, coordination, and operational preparedness. Addressing these interconnected system-level deficiencies is likely to enhance the resilience and responsiveness of health systems managing cholera outbreaks in humanitarian settings.

Our analysis was limited to two refugee sites, focusing primarily on human factors contributing to the cholera outbreak. However, other potential mediating factors were not examined in this study. For instance, environmental variables such as climate change, geographical topology, and shifting rainfall patterns may have influenced cholera transmission. Existing research suggests that these factors can significantly contribute to the spread of cholera, but their specific impact in this context was beyond the scope of our analysis. This study also included only individuals who had recovered from cholera; therefore, the findings may be subject to survivorship bias, as the experiences of patients who died or experienced the most severe outcomes were not captured. Future studies should incorporate these elements to provide a more comprehensive understanding of cholera dynamics in similar settings.

## Conclusion

This research topic integrates the STAMP-CAST methodology into the study of cholera outbreaks and response, offering a systems thinking perspective to enhance the understanding and effectiveness of response strategies in the unique context of refugee settings. Our findings support the adoption of a differentiated approach to strengthening cholera interventions by both local institutions (e.g., Kenya’s Ministry of Health and the Center for Disease Control) and global organizations. This aligns with the GTFCC mission to establish an effective framework for coordinated technical support, financing, and resource allocation at both global and national levels. By examining control structures, feedback mechanisms, and interactions among multiple actors, the study provides insights into systemic factors influencing outbreak preparedness and response [1, 58]. The findings highlight the importance of strengthening governance, surveillance, coordination, community engagement, and WASH systems to improve resilience against future cholera outbreaks.

## Supplementary Information

Supplementary Material 1.

## Data Availability

The datasets generated and/or analyzed during the current study are available from the corresponding author on reasonable request.

## Abbreviations

CDC: Center for Disease Control
CHWS: Community Health Workers
CTC: Cholera Treatment Centers
GTFCC: Global Task Force on Cholera Control
MOH: Ministry of Health
MRC: Medical Research Council
SOP: Standard Operating Procedures
STAMP-CAST: Systems Theoretic Accident Model and Process- Causal Analysis based on Systems Theory
TWG: Technical Working Group
WASH: Water, Sanitation, and Hygiene
WHO: World Health Organization
