# Supplementary material for: Cholera endemicity as a systems failure: a STAMP-CAST investigation in Kakuma and Dadaab refugee complexes in Kenya
Source: BMC Public Health. 2026 Aug 7;26:3195. doi: 10.1186/s12889-026-28948-z (PMC13733060; doi:10.1186/s12889-026-28948-z)
Supplement: Supplementary file 1 [file 12889_2026_28948_MOESM1_ESM.pdf]

Additional File 1

Supplementary Material

Title: Cholera Endemicity as a Systems Failure: A STAMP-CAST Investigation in Kakuma and Dadaab Refugee Complexes in Kenya

Authors:

James Oyoo[1], Annette Murunga[1], Kevin Otieno[1,2], Ben Ngoye[1]

Affiliations:

[1]Strathmore University Business School, Institute of Healthcare Management, Nairobi, Kenya

[2]Strathmore Institute of Mathematical Sciences, Nairobi, Kenya

Country Affiliation:

All authors are from Nairobi, Kenya

Corresponding Author:

James Oyoo

Email: joyoo@strathmore.edu

Strathmore University Business School, Institute of Healthcare Management, Nairobi, Kenya

Supplementary Figures

This supplementary file contains the detailed actor maps used to construct the STAMP-CAST system control structure presented in the main manuscript. These figures provide the complete representation of organizations, governance levels, and interactions identified through document review, stakeholder mapping, key informant interviews, focus group discussions, and policy analysis.

Contents

Supplementary Figure S1.
Actor map of the cholera outbreak response system in **Kakuma Refugee Camp**, Turkana County, Kenya.

Supplementary Figure S2.
Actor map of the cholera outbreak response system in **Dadaab Refugee Camp**, Garissa County, Kenya.

Description

The actor maps illustrate the organizations and stakeholders involved in cholera outbreak preparedness and response across multiple governance levels. They informed the development of the STAMP-CAST control structure presented in the main manuscript by identifying system controllers, control actions, communication pathways, and feedback mechanisms operating within the humanitarian response system.

SYSTEM LEVEL	ACTORS MAP: TURKANA COUNTY
1. International Bodies, Policy Guidance & Resourcing	USAID-OFDA; IGAD; ICRC; WHO; UNICEF; IFRC; IOM; UNHCR; International Law On Refugee Management; CDC; WFP; WHH
2. National Government, Policy & Legislative, Guidance, Coordination, Territorial Integrity	COG; Inter- Ministerial Committees; Ministry of Education; Parliamentary Committee on Health; Ministry of Transport & Infrastructure Development; The National Assembly; Northern Corridor Development Authority; National Emergency Response Committee; MOH; Ministry of Agriculture; Ministry of Devolution & ASAL Areas; The National Treasury; Refugee Affairs Committee on Health; Ministry of Water Sanitation & Irrigation; The Senate; Ministry of Interior; NOREB
3. Regulatory Bodies, National Agencies, State Departments	Port Health; Research Centers (e.g. KEMRI)); WHO-Kenya Country Office; K-FELTP; Professional Health Bodies Regulators e.g. COC, KMPDC, PPB, KMTTB, Nursing Council; Mainstream Media; CDC-Kenya; Health Training Institutions (e.g. KMTC, Universities); Refugee Consortium
4. County Government & Other Camp Actors	MSF; DRC; IRC; LOKADO; LWF; AMREF; KRCS; NCCK; CARE; DRS; Team and Team; Refugee Consortium; Turkana County Government; AIC – Health Ministries; World Vision; Diocese of Lodwar; County Steering Group; PWJ
5. Technical and Operational, Management Decisions & Actions	Refugee Management Committee; WASH; Host Community Groups; Social Media; Food Premises; Technical Working Group on Cholera Outbreak; Refugee Households; Schools & Other Learning Institutions; Cholera Treatment Centers
6. Physical Resources & Actor Decisions & Actions	Transit / Reception Centers; Host community; New Refugees; Returning Refugees; Economic Activities & Resources
7. Environment & Equipment	Roads; Water and Sewerage Services; Waste disposal; Housing; Food

SYSTEM LEVEL	ACTORS MAP: GARISSA COUNTY
1. International Bodies, Policy Guidance & Resourcing	USAID-OFDA; IGAD; ICRC; WHO; UNICEF; WHH; IOM; UNHCR; GIZ; CDC; WFP
2. National Government, Policy & Legislative, Guidance, Coordination, Territorial Integrity	COG; Inter- Ministerial Committees; Ministry of Education; Parliamentary Committee on Health; Ministry of Transport & Infrastructure Development; The National Assembly; Northern Corridor Development Authority; National Emergency Response Committee; MOH; Ministry of Agriculture; Ministry of Devolution & ASAL Areas; The National Treasury; Refugee Affairs Committee on Health; Ministry of Water Sanitation & Irrigation; The Senate; Ministry of Interior; PHEOC
3. Regulatory Bodies, National Agencies, State Departments	Port Health; Research Centers (e.g. KEMRI)); WHO-Kenya Country Office; K-FELTP; Department of public health & Sanitation; Lab Regulation (KMTTB and KMLTB); Department of Urban Planning; Mainstream Media; CDC-Kenya; Health Training Institutions (e.g. KMTC, Universities); Refugee Consortium; MOH - Disease Surveillance and Response Unit.; Professional Health Bodies Regulators e.g COC, KMPDC, PPB, KMTTB, Nursing Council
4. County Government & Other Camp Actors	MSF; American Refugee Council; IRC; Film Aid; LWF; AMREF; KRCS; COEC; CARE; PWJ; DRS; Multi-Agency Response Teams; County Assembly; Garissa County Government; County & Sub-County Outbreak Response Teams; World Vision; SUPKEM & Other Religious Bodies; County Steering Group
5. Technical and Operational, Management Decisions & Actions	Water, Environment & Sanitation Coordination (WESCOORD) Committee – (Chaired by the CPHO); Community Groups (CHU, Water User Committees); Food Premises; Social & Mainstream Media; Schools & Other Learning Institutions; Cholera Treatment Centers; County/Sub-County WASH Committee; Refugee Management Committees/ Block & Section Leaders; Refugee Households; Host Community Leadership
6. Physical Resources & Actor Decisions & Actions	Transit / Reception Centers; Host community; New Refugees; Returning Refugees; Economic Activities & Resources
7. Environment & Equipment	Roads; Water and Sewerage Services; Waste disposal; Housing; Food; HRH; Essential Medical Supplies & Commodities
